# Epithelial cell alarmin cytokines: Frontline mediators of the asthma inflammatory response

**DOI:** 10.3389/fimmu.2022.975914

**Published:** 2022-10-14

**Authors:** Marc Duchesne, Isobel Okoye, Paige Lacy

**Affiliations:** Alberta Respiratory Centre, Department of Medicine, University of Alberta, Edmonton, AB, Canada

**Keywords:** TSLP, IL-25, IL-33, tezepelumab, allergy

## Abstract

The exposure of the airway epithelium to external stimuli such as allergens, microbes, and air pollution triggers the release of the alarmin cytokines IL-25, IL-33 and thymic stromal lymphopoietin (TSLP). IL-25, IL-33 and TSLP interact with their ligands, IL-17RA, IL1RL1 and TSLPR respectively, expressed by hematopoietic and non-hematopoietic cells including dendritic cells, ILC2 cells, endothelial cells, and fibroblasts. Alarmins play key roles in driving type 2-high, and to a lesser extent type 2-low responses, in asthma. In addition, studies in which each of these three alarmins were targeted in allergen-challenged mice showed decreased chronicity of type-2 driven disease. Consequently, ascertaining the mechanism of activity of these upstream mediators has implications for understanding the outcome of targeted therapies designed to counteract their activity and alleviate downstream type 2-high and low effector responses. Furthermore, identifying the factors which shift the balance between the elicitation of type 2-high, eosinophilic asthma and type-2 low, neutrophilic-positive/negative asthma by alarmins is essential. In support of these efforts, observations from the NAVIGATOR trial imply that targeting TSLP in patients with tezepelumab results in reduced asthma exacerbations, improved lung function and control of the disease. In this review, we will discuss the mechanisms surrounding the secretion of IL-25, IL-33, and TSLP from the airway epithelium and how this influences the allergic airway cascade. We also review in detail how alarmin-receptor/co-receptor interactions modulate downstream allergic inflammation. Current strategies which target alarmins, their efficacy and inflammatory phenotype will be discussed.

## Asthma: A heterogenous response in the airway epithelium

Asthma is a multifactorial, diverse, and heterogenous malady comprising of a combination of respiratory symptoms, which stem from chronic airway inflammation and tissue remodelling. Early accounts of asthma symptoms can be traced back to the 4^th^ century BC, when it was described in the Hippocratic Corpus as spasms of breathlessness observed mainly in metalworkers, anglers, and tailors (1, 2). Recently, clinicians and researchers have sought to redefine asthma as a collection of chronic airway manifestations which vary, depending on treatable symptoms (such as airflow limitation, eosinophilic inflammation, and comorbidities), and factors including age, lifestyle, and environment (3). Other recent approaches based on systems biology techniques have classified asthma based on T2 (type 2) high and non-T2 high endotypes designated according to cellular mechanisms and molecular pathways [reviewed in (4)].

**Figure 1:**
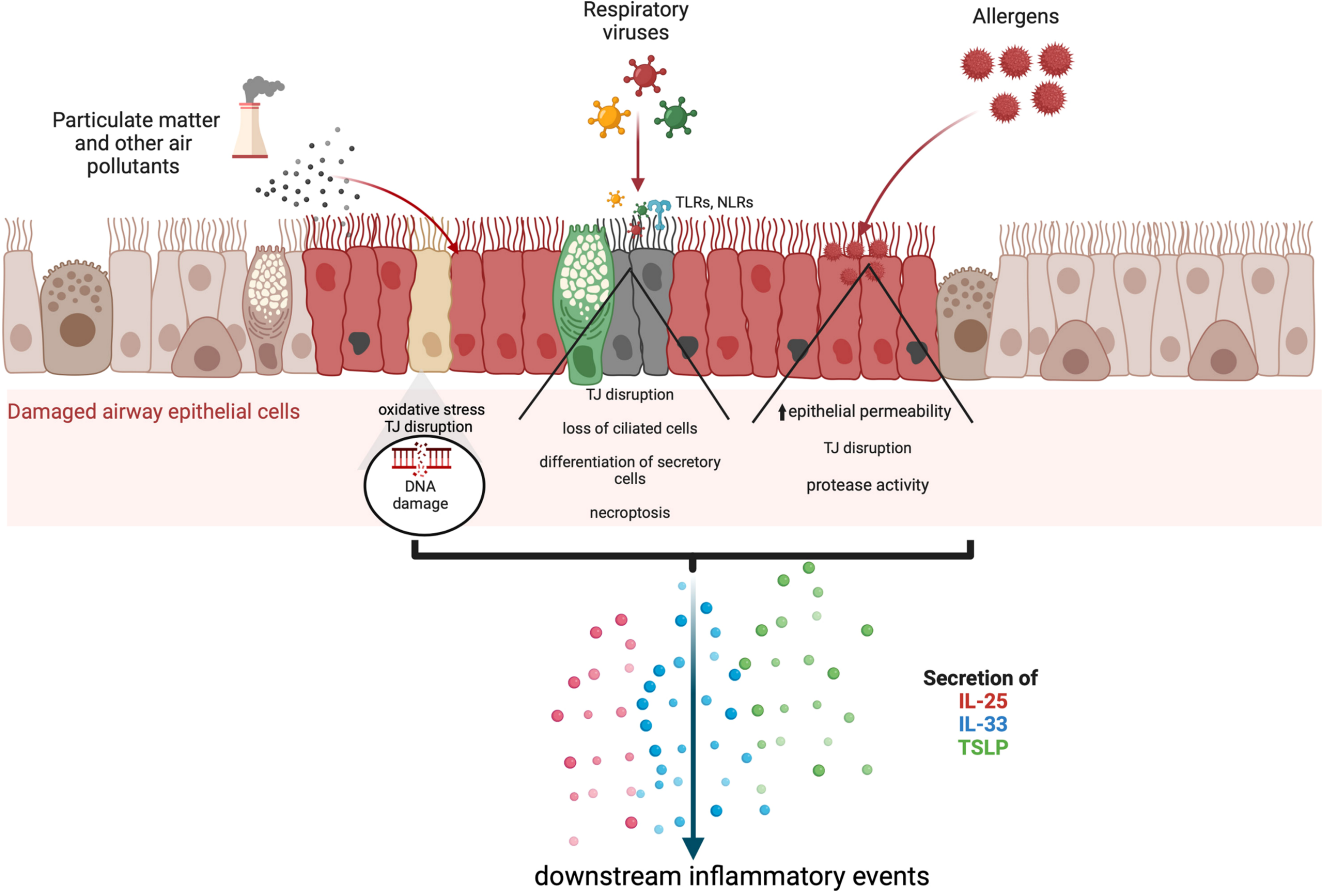
The secretion of alarmin cytokines in the airway epithelium may be triggered through multiple mechanisms which depend on the nature of the external insult. Asthma exacerbations can occur in response to air pollutants, allergens, and respiratory viruses. Various studies based on in vitrochallenged human bronchial epithelial cells and animal models indicate that IL-25, IL-33 and TSLP are released through cleavage of tight junctions (TJ), and necroptosis. Particulate matter and other air pollutants can trigger oxidative stress and the release of reactive oxygen and nitrogen species as well as DNA damage.

Alarmin cytokine production in asthma may be initiated by a wide spectrum of triggers including multiple allergens, microbes, and air pollutants, which can promote loss of integrity as well as anchorage of the respiratory epithelium as shown in **Figure 1** (4, 5). Allergic, or atopic, asthma is thought to be established by a sensitisation phase to allergens, followed by a challenge phase which initiates airway hyperreactivity. These environmental perturbations can induce alarmin production from epithelial cells, which facilitate downstream infiltration of inflammatory mediators and events leading to airway hyperreactivity. In this review, we cover the triggers and effects of alarmin cytokines released from airway epithelial cells and summarise how these may be targeted for therapeutic intervention.

### Allergens

The onset of asthma and its exacerbations may be triggered by inhalation of allergens such as house dust mite (HDM) allergens, cat dander, cockroach allergens, and *Aspergillus* spores. It is noteworthy that allergens originate from organisms that are not inherently harmful to the host. Allergens consist mainly of proteins with functional domains that confer enzymatic and/or ligand-binding activity, which inadvertently facilitate the interaction and modification of airway epithelial cells. Consequently, the nature of immune responses elicited by allergen sensitisation and challenge depends on the properties of the relevant allergen as well as genetic polymorphisms in the allergic individual that are thought to predispose individuals to asthma.

The ability of allergens to differentially induce asthma in patients may reflect several factors including the underlying mechanisms of epithelial cell interaction and disruption. For example, the cockroach allergen Per a 10 increases secretion of IL-33 and TSLP *in vitro* (6). Of relevance is the elevation of allergic inflammation, including increased IL-33, TSLP, IL-1α and uric acid in the lungs of Per a 10-treated mice (6). Interestingly, the intrinsic protease activity of some allergens is responsible for eliciting alarmin cytokine production. The serine protease activity of the fungus *Alternaria alternata* evokes secretion of IL-33 in airway epithelial cells of challenged mice, a mechanism dependent on the activation of protease activated receptor-2 and ATP signaling (7). These findings suggest that various enzyme activities in allergens may induce alarmin cytokine expression and can impact downstream allergic airway responses.

Furthermore, TSLP secretion is increased by lung epithelia exposed to carbohydrate-dependent natural Der p1. This was confirmed by minimal uptake of deglycosylated Der p1 by DCs (8). Thus, glycosylation of allergens can also have a significant impact on their allergenic properties. These observations are indications of various biochemical mechanisms harnessed by allergens, which contribute to alarmin secretion and downstream airway inflammatory responses.

### Virus infections

Viruses can induce epithelial disruption, and studies in mice have demonstrated that viral infections can promote the secretion of alarmin cytokines such as IL-33 by non-hematopoietic cells and necrotic cells (9, 10). IL-33 signalling through its receptor, ST2 expressed by cytotoxic CD8^+^ T cells, can promote the expansion of primary effector CD8^+^ T cells and the differentiation of cytotoxic T cells in LCMV-infected mice (10). Furthermore, IL-33 release can modulate innate and adaptive responses to HIV, viral hepatitis and HSV infections (11). Importantly, IL-33 has been implicated in COVID-19-associated lung remodelling, fibrosis, anti-viral cytotoxic T cell activity, antibody production and release (12, 13).

### Air pollution

Airborne particulate matter includes solid particles or air-suspended liquid droplets of various sizes (in diameters), which can be differentially deposited and absorbed in the airways and thereby induce local inflammation and asthma exacerbation. Studies have shown that the expression of TSLP, IL-25 and IL-33 as well as other Th2 cytokines are increased in particulate matter-exposed cells (reviewed in (14). However, few studies have shown how oxidative stress, DNA damage and increased arginase II activity directly impact TSLP, IL-25 and IL-33 secretion in airway epithelial cells. There is the likelihood that similar to results from studies on HDM-induced asthma, the secretion of alarmin cytokines in response to particulate matter-associated DNA damage will depend on the levels of DNA double-strand breaks, repair proteins and apoptosis of airway epithelial cells (15). A time-dependent increase in the production of arginase II observed in particulate matter-treated bronchial epithelial cells (16) may be an indication of upstream alarmin cytokine secretion and amplified type 2 responses.

Other studies have focused on the correlates of particulate matter-induced oxidative stress (17, 18). TSLP secretion has been found to occur in response to direct epithelial cell injury, particularly during tight junction disruption by particulate matter (14, 19). In a proof-of-concept study in which human bronchial epithelial cells were co-cultured with myeloid DCs, treatment of co-cultures with diesel exhaust particles upregulated OX40 ligand and Jagged-1 expression by DCs, which was dependent on TSLP release (20, 21). In a study set up to investigate the role of oxidative stress in response to allergens, IL-33 release was found to be controlled by the transcription factor nuclear factor-erythroid-2-related factor 2 (Nrf2) (22). In another study by Brandt et al., the authors found that exposure to diesel exhaust particles promoted oxidative stress, IL-6, neutrophils and pulmonary accumulation of IL-33; however, IL-25 and TSLP levels were unaffected (23).

The expression of the aryl hydrocarbon receptor (AhR) by bronchial epithelial cells promotes mucin production and enhanced allergic responses (24). A link has been identified between TSLP upregulation and expression of miR-375, which targets the AhR (25). Results from another study show increased secretion of IL-25, IL-33 and TSLP in primary bronchial epithelial cells in response to with diesel exhaust particles treatment, which was reversed by silencing AhR expression (26). Results from chromatin immunoprecipation assays indicated that binding of the AhR nuclear translocator to the IL-33, IL-25 and TSLP promoters facilitated severe allergic manifestations (26).

### Alarmin cytokines: Heralds of epithelial stress

**Figure 2:**
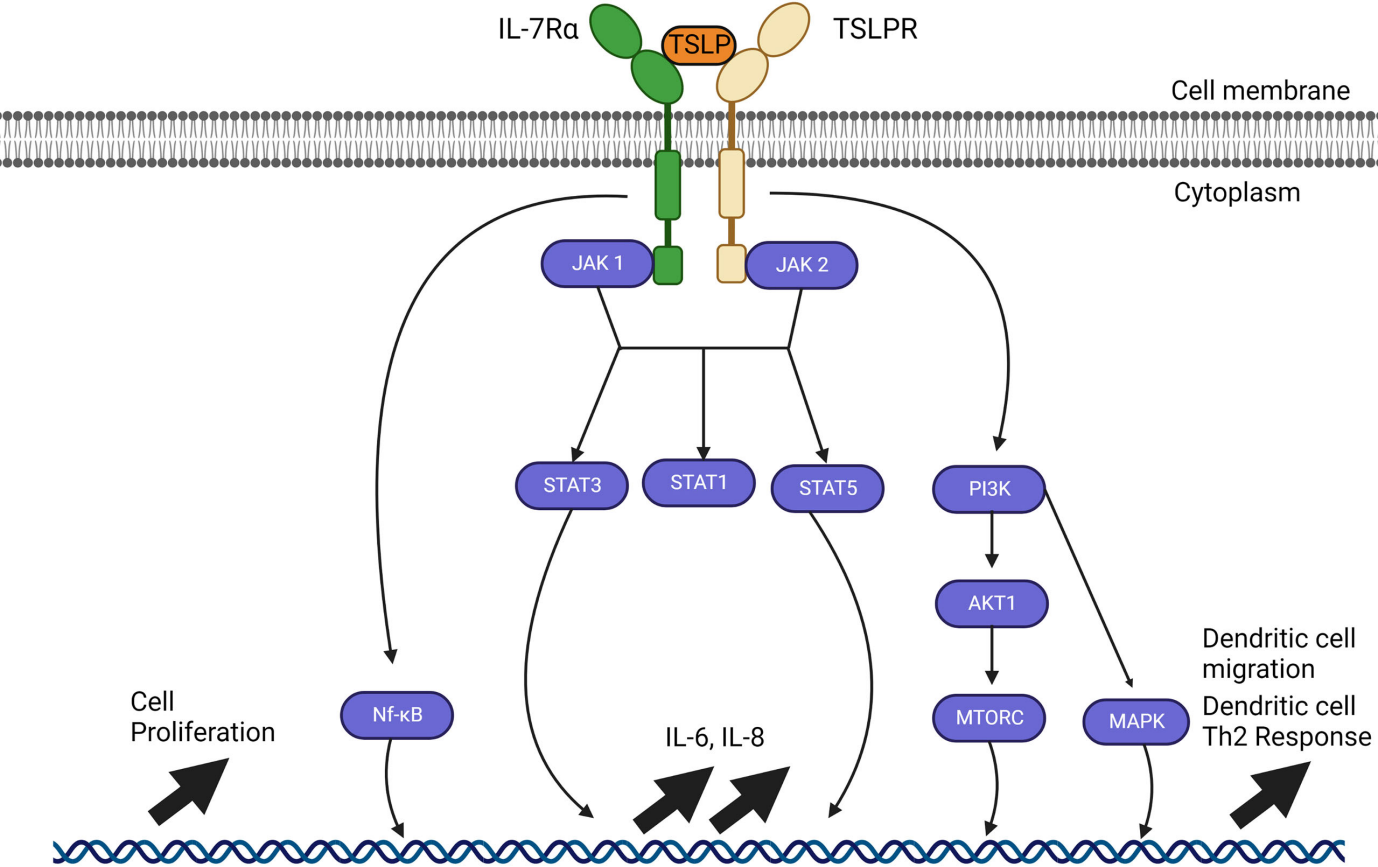
TSLP signalling pathways through JAK1 and JAK2. TSLP signaling pathways require a heterodimer of IL-7Ra and TSLP receptor (TSLPR). Interaction between TSLP and its receptor cascades in a signaling pathway through JAK1 and JAK2 which varies depending on the cell type. JAK1 and JAK 2 can activate STAT1, STAT3 and STAT5 as well as NF-kB and MAP kinases through PI3K, but all these pathways have not been fully resolved. The outcome of activation also varies with the type of cell affected, but includes upregulation of IL-6, IL-8, cell proliferation; in dendritic cells, activation by TSLP causes their migration and maturation to a Th2 phenotype.

**Figure 3:**
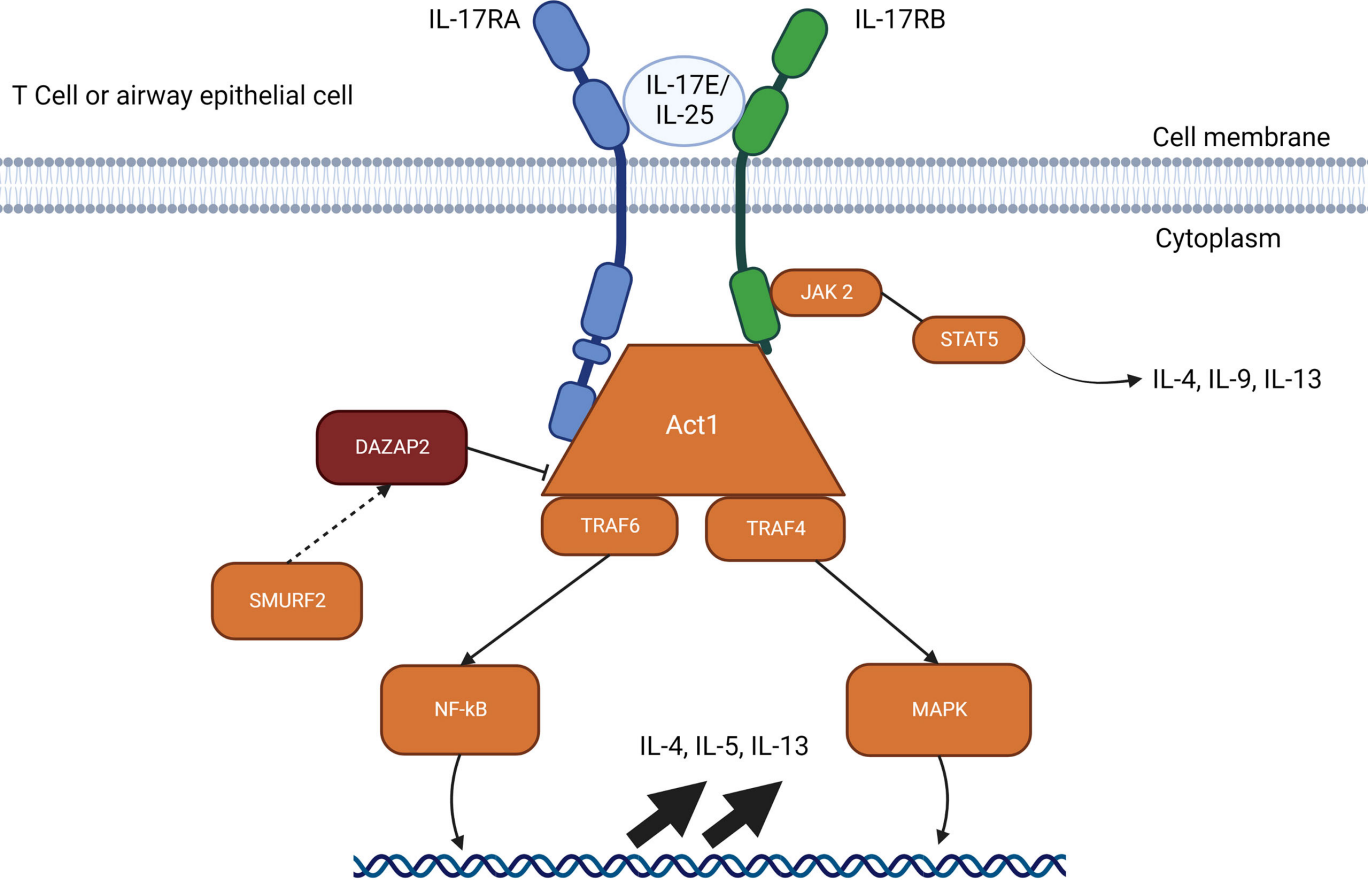
IL-25 signalling pathways through Act1. The IL-17E/IL-25 signaling pathway requires a heterodimer of IL-17RA and IL-17RB which allows interactions with Act1. Act1 recruits TRAF4 and TRAF6 which activates gene upregulation through NF-kB. SMURF2 removes the inhibitory effects of DAZAP2 which allows upregulation through MAP kinases and induces JAK2 to signal through STAT5 for IL-4, IL-9 and IL-13. The IL-25 pathways available represent the resolved pathways in T cell and airway epithelial cells, but IL-25 receptors are also found on mast cells, eosinophils, basophils, and other immune cells.

**Figure 4:**
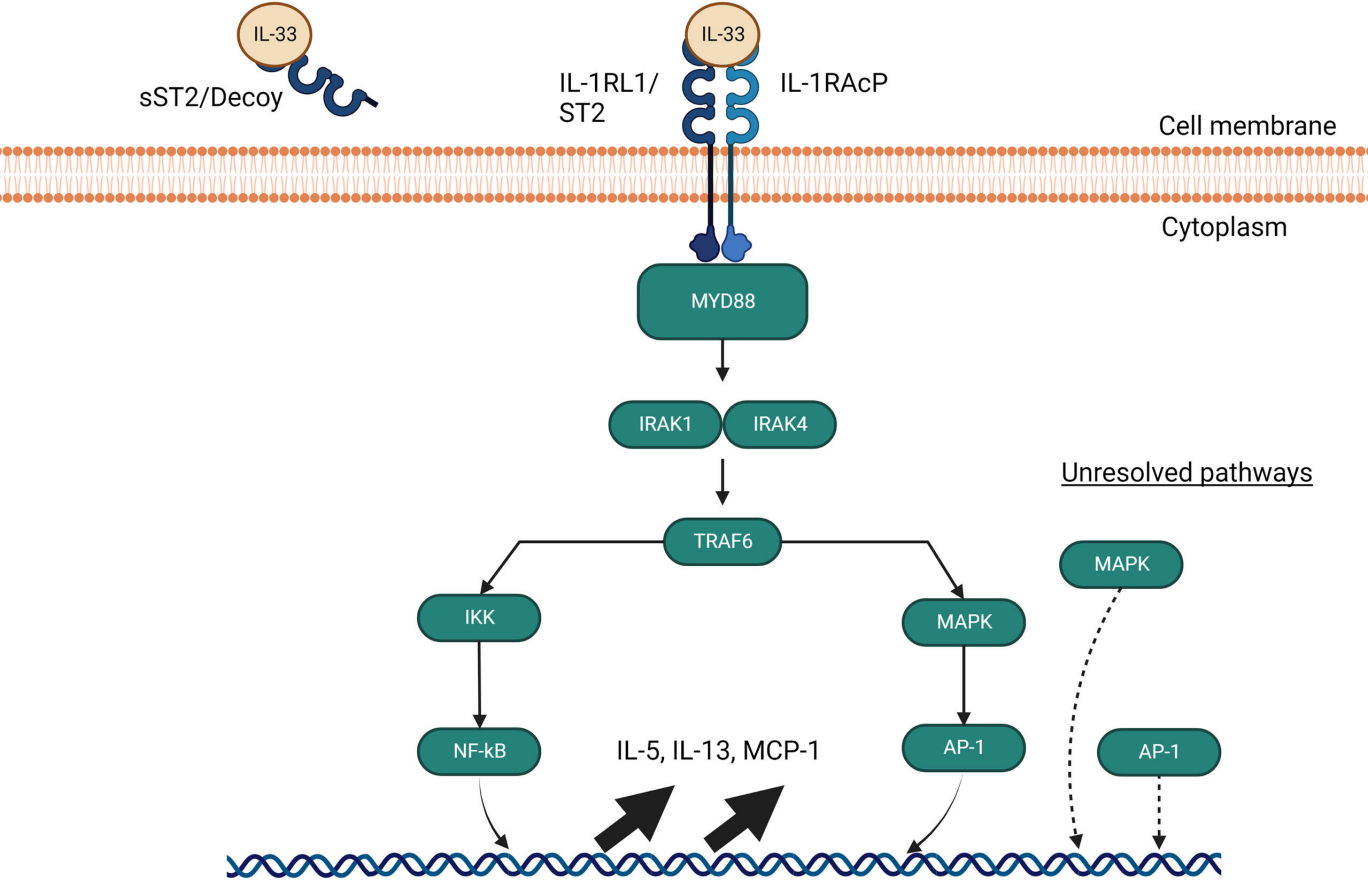
IL-33 signalling pathways through MyD88. The IL-33 signaling pathway requires a heterodimer of ST2 and IL-1 receptor accessory protein (IL- 1RAcP) to activate and signal through MyD88. If IL-33 avoids binding to soluble ST2 decoys, it signals through MyD88 to interact with IRAK1 and IRAK4, which in turns activate TRAF6 and leads to NF-kB and AP-1 gene upregulation of IL-5, IL-13 and MCP-1. AP-1 is also able to activate independently of MyD88 through an undiscovered pathway involving MAP kinases.

Alarmin cytokines are a small group of epithelial-derived mediators of the immune system holding a pivotal role in initiating T2 inflammatory responses in asthma. These mediators correspond to TSLP, IL-25, and IL-33, in the order of their discovery (27–30). Their characterizations as alarmins signify that these cytokines possess traits associated with other alarmins: they can be produced by healthy activated immune cells and secreted through the ER-Golgi apparatus or non-classical pathways, such as nonprogrammed cell death, but not apoptosis; they promote the adaptive immune system directly or indirectly through the recruitment and activation of antigen-presenting innate immune cells such as DCs and ILC2; and they can promote homeostatic functions such as tissue repair and immune system processes (29, 31–35). Despite their similar roles, their signaling pathways differ greatly between them depending on the cell type, as shown in **Figures 2**–**4**.

Signalling pathways triggered in response to allergens and/or pollutants influence downstream inflammatory responses that underlie the complex nature of asthma endotypes. Of importance is the ability of the alarmin cytokines TSLP, IL-25 and IL-33 secreted by epithelial cells to activate T2 immune responses, which are mediated by T helper 2 (Th2) and group 2 innate lymphocyte cells (ILC2). T2 airway inflammation is characterised by allergic and non-allergic eosinophilic inflammation, driven by IL-5 produced by Th2 and ILC2 cells (36). TSLP has also been implicated in the activation of non-T2 or T2-low airway responses driven by T helper 17 cells and neutrophilic inflammation (36). In addition, TSLP can regulate the interaction between airway smooth muscle cells and mast cells and promote structural changes that trigger airway remodelling (37–39). Collectively, it is evident that from their prime position in the inflammatory cascade, TSLP, IL-25 and IL-33 orchestrate the elicitation and activity of multiple effector cells and pathways that constitute the asthma phenotype (**Figure 1**).

The pivotal role of alarmin cytokines has generated substantial interest in their respective functions; however, due to the pleiotropic nature of these cytokines, fully understanding their roles within the immune system has been difficult. Here, we try to deconstruct their roles from research over the last twenty years.

#### Thymic stromal lymphopoietin (TSLP) structure and function in the airways

The originally characterised alarmin cytokine TSLP, a member of the IL-2 family of cytokines, was first identified as a B cell growth factor in a mouse model in 1994 (40). Later classified as an IL-7-like cytokine, its receptor is a heterodimer comprised of the IL-7 receptor α chain (IL7Ra) and a g-like receptor chain called TSLP receptor (TSLPR) (41). TSLPR is located on various immune cell types, which include immature DCs, mast cells, activated CD4^+^ T cells, CD8^+^ T cells, B cells, NKT cells, basophils, eosinophils, and monocytes, with the highest expression in myeloid DC populations (41–46). This feature also highlighted that TSLP has multiple signaling pathways which varies by cell type that remain to be solved (47). Unlike its receptor, TSLP expression is restricted with its primary expression site in airway epithelial cells, extending to airway smooth muscle cells and mast cells in asthma, and activating a caspase-1/NF-κB pathway (37, 48, 49). The wide distribution of TSLPR expression indicates the potency of TSLP to induce broad effects throughout the body, although much remains to be elucidated regarding the role of TSLP in airway inflammation and asthma.

TSLP is a pleiotropic cytokine capable of inducing both pro-inflammatory and regulatory functions within the immune system. Early studies in mouse models demonstrated a necessary and sufficient role for TSLP in the initiation of allergic inflammatory diseases based on transgenic lung-specific expression of TSLP in antigen-induced asthma and similar models in TSLPR-deficient mice (50, 51). Antibodies to TSLP were subsequently found to reduce the inflammatory response and airway remodeling in mouse models of chronic allergic airway inflammation (52). This led to the concept of targeting TSLP in human asthma at the level of the airway epithelium.

**Figure 5:**
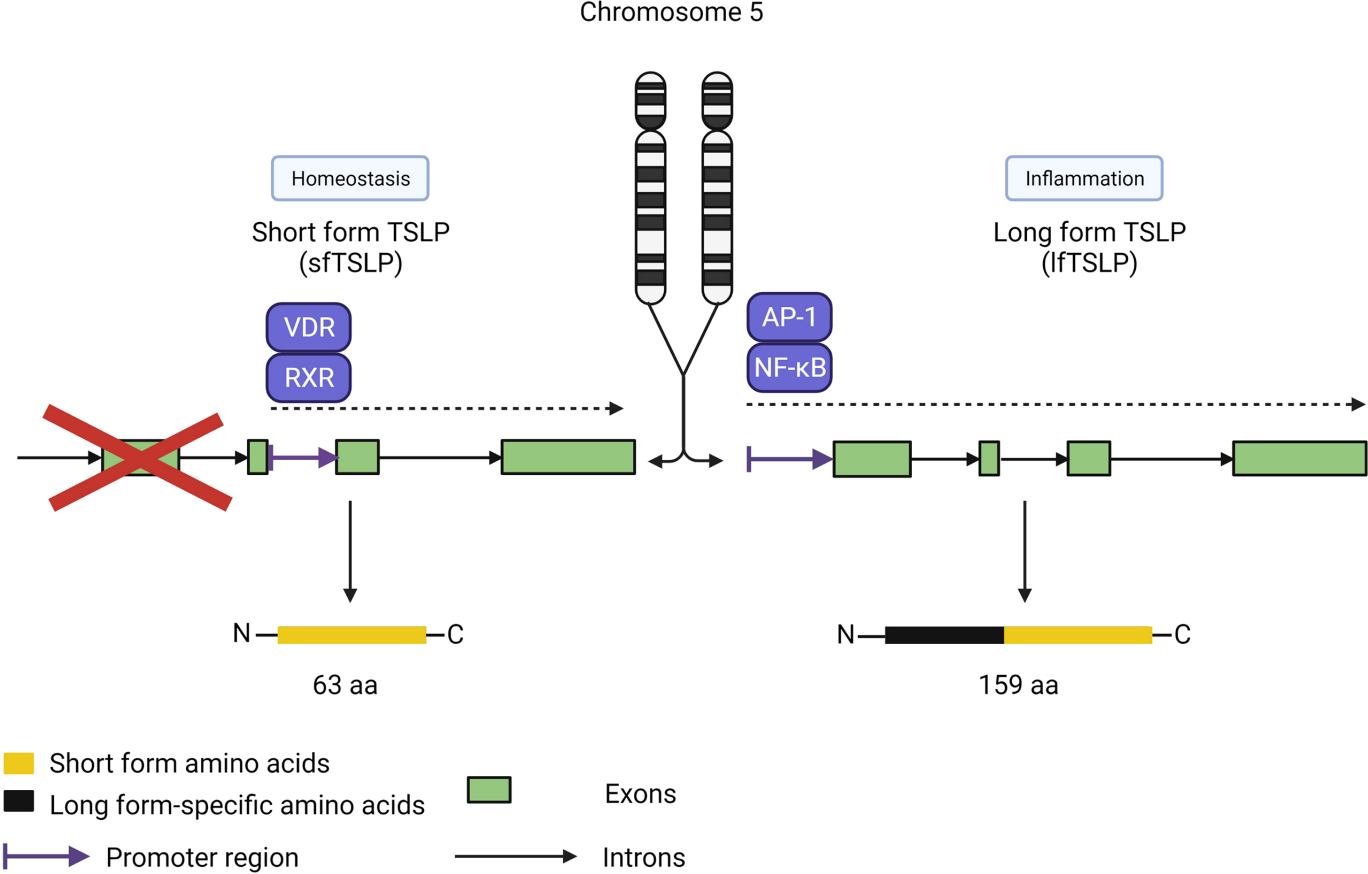
TSLP isoforms. TSLP has two isoforms covering two distinct roles: sfTSLP (63 amino acids) for homeostasis and lf TSLP (159 amino acids) for inflammatory responses. Each isoform is under the control of different promoter regions directed by distinct pathways, with the vitamin D receptor/retinoid X receptor pathway specific for sfTSLP expression and the AP-1/NF-kB pathway for lfTSLP. Both isoforms share the same Cterminus amino acid sequence but have distinct N-terminus sequences.

Interestingly, the functions of TSLP are uniquely split between two isoforms in humans, with short-form TSLP (sfTSLP) associated with promoting homeostasis and long-form TSLP (lfTSLP) related to promoting airway inflammation (**Figure 5**) (53–55). An additional complexity of these isoforms is the human-specific expression of sfTSLP, with no evidence for its expression in rodents or other species (56). The two isoforms were first described in human bronchial epithelial cells by Harada et al. (53). While human lfTSLP consists of 159 amino acids, sfTSLP is considerably shorter at 63 amino acids, which are identical in the C-terminal region of lfTSLP. These proteins derive from transcripts produced from two distinct 5’-untranslated regions resulting in two different open reading frames for TSLP in the human genome (57). The expression and release of these two isoforms are differentially regulated; stimulation of keratinocytes by T2 cytokines or ligands for TLRs resulted in increased lfTSLP transcription but not sfTSLP (58). In contrast, vitamin D upregulated total, but not long form, TSLP transcription, although it did not induce the release of TSLP, suggesting that sfTSLP may be regulated by vitamin D.

Comprehensive studies in mice have elucidated the role of mouse TSLP (similar to human lfTSLP) in the development of asthma and other type 2 inflammatory manifestations, represented by the induction of downstream responses by TSLPR-expressing cells such as DCs, ILC2s, basophils and mast cells (59). However, the lack of sfTSLP expression in mice makes it difficult to compare the specific immunological role of sfTSLP with that of lfTSLP in homeostasis or allergic inflammation in a whole animal model.

Expression of lfTSLP is highly inducible by many cytokines in bronchial epithelial cells, suggesting a proinflammatory role for this isoform in the lungs (53), and a recent study showed elevated sfTSLP and lfTSLP mRNA expression in nasal epithelial cells but not the circulating blood of children with asthma (55). Such localized expression of these isoforms suggests that they have important homeostatic and proinflammatory roles at the level of the tissue microenvironment rather than systemic circulation.

Studies reveal several allergen-derived proteases such as *Alternaria*, Per a 10 in cockroach extract, and Der-p2 from HDM can induce TSLP, although these studies did not discriminate between sfTSLP and lfTSLP (6, 60–63). Additionally, respiratory viruses, bacterial peptidoglycans, double-stranded RNA (dsRNA), cigarette smoke, and some pro-inflammatory cytokines such as IL-1, IL-4, IL-13, and TNF-a and can induce TSLP in epithelial cells and immune cells, ultimately promoting airway inflammation (37, 43, 48, 64–67). Air pollutants such as diesel exhaust particles can also amplify TSLP release triggered by allergens, which links decreasing air quality in heavily urbanised areas with an increased susceptibility of asthma and asthma exacerbation in urban centers.

The central role of TSLP in airway inflammation is to activate CD11c^+^ myeloid DCs to express OX40 ligand (OX40L), which then primes naïve CD4^+^ T cells to differentiate into a pro-inflammatory T2 phenotype expressing IL-4, IL-5, IL-13, and TNF-a (41, 43, 44, 48, 49). T regulatory cells interacting with TSLP-activated DCs (through OX40L) switch from an IL-10 producing regulatory subtype into a pro-inflammatory TNF-a producing subtype (41). When activated, CD4^+^ T cells are maintained and expanded through TSLP-activated DCs expressing the prostaglandin D2 receptor CRTH2, which contribute to the proliferation of Th2 memory cells. Tissue damage is also promoted directly through TSLP-activated DCs by activating naïve CD8^+^ T cells and inducing their differentiation into an ineffective cytotoxic cell with prominent IL-5 and IL-13 producing capabilities. These cells can be further amplified by the presence of CD40L, which induces differentiation of CD8^+^ T cells into cytotoxic effector cells that can produce IL-5, IL-13, and a large amount of IFN-γ (41, 43, 44, 48, 49, 64, 66, 68).

While DCs remain the primary target cell type for TSLP, other TSLPR-expressing cells of the innate immune system also contribute to T2 cytokine-mediated allergic responses initiated by TSLP. For example, NKT cells expressing TSLP heterodimers are directly activated by TSLP to produce IL-13 (69). Eosinophils are particularly affected by TSLP, since eosinophils interacting with TSLP survive for a significantly longer time before undergoing apoptosis through the expression of an increased amount of adhesion molecules that bind to fibronectin, which then further enhances their survival (70, 71). TSLP also induces release of IL-6 and the chemokines CXCL1, CXCL8, and CCL2 from eosinophils, as well as promoting the migration of neutrophil and non-hematopoietic cells (44, 70).

Mouse model studies suggest that eosinophilia and basophilia are promoted through TSLP-mediated upregulation of CD34^+^ progenitor cells and their recruitment at sites of infections (72, 73). Secreted cytokines from CD34^+^ were shown to activate epithelial cells and promote TSLP release in a positive feedback loop, while TSLP induced differentiation of CD34^+^ cells into eosinophils and basophils and further enhanced allergic inflammation in the tissue (40, 72, 74). Other studies in humans found similar properties of TSLP in mediating basophil and eosinophil hematopoiesis, skewing the immune system towards a T2 high phenotype (41, 72–75). Findings from these studies also revealed that TSLP promotes the formation of extracellular traps from eosinophils and regulates basophil degranulation, which then initiates a positive feedback loop of TSLP production and exacerbates allergic airway inflammation (71, 72, 74, 75). Interestingly, eosinophil extracellular trap induction by TSLP is associated with the severity of asthma and promotes this through pulmonary neuroendocrine cells *via* the CCDC25-ILK-PKCα-CRTC1 pathway (76). However, there are no studies showing how TSLP itself is secreted from epithelial cells, although one study has indicated both apical and basolateral secretion pathways are possible for TSLP from bronchial epithelial cells (77).

On its own, TSLP is a powerful inducer of airway inflammation, but it can also work synergistically with two alarmin cytokines, IL-25 and IL-33. Studies have shown that IL-33 and TSLP modulate migration of CD34^+^ progenitor cells in patients with asthma, further enhancing eosinophilia and basophilia, and also inversely correlate with lung function (78). For its part, IL-25 mainly affects TSLP by its contribution to Th2 memory cell expansion through proliferation, T2 polarization, and its effect on upregulation of chemokine and cytokine receptors, CCR4, IL-4Rα, and IL-7Rα (79). Asthmatic patients exhibited an increase in cells immunoreactive for IL-25, IL-33, and TSLP in their submucosal lung samples, which was accompanied by increased airway obstruction characterised by late-phase bronchoconstriction and reduced FEV_1_ following allergen exposure (34). These findings suggest that TSLP can work both independently and synergistically with other alarmin cytokines to contribute to the pathogenicity of allergic airway inflammation (30, 80–82).

#### Function of interleukin-25 (IL-25) in the airways

IL-25, also known as IL-17E, belongs to the IL-17 cytokine family, including IL-17A, IL-17B, IL-17C, IL-17D, and IL-17F (30, 80–82). IL-25 was discovered in 2001 in a mouse model study showing that infusion of IL-25 induced a T2-like response (83). In the airways, IL-25 is secreted by airway endothelial cells and epithelial cells, as well as immune cells such as alveolar macrophages, mast cells, Th2 cells, activated eosinophils, and basophils (35, 81, 84–90). The widespread expression of IL-25 in numerous tissue and immune cells highlights the broad impact of this cytokine in both the initiation and progression of allergic inflammation.

The receptor for IL-25 is a heterodimer complex composed of the two subunits, IL-17RA and IL-17RB (88). After formation of the heterodimer complex, IL-25-mediated signaling pathway recruits the adaptor protein Act 1, followed by TRAF4 and TRAF6. TRAF6 activates the NF-κB pathway while TRAF4 activates the MAPK pathway through degradation of the inhibitory protein DAZAP2 (deleted in azoospermia DAZ-associated protein 2) by Smad ubiquitin regulatory factor 2 (SMURF-2) (30, 87, 91, 92). The currently understood signaling pathway for IL-25 (**Figure 3**) is still under investigation and requires further study to identify nuances between various cell types.

The immunological role of IL-25 may be associated with protection against parasitic infections. This is supported by the observation that epithelial-derived IL-25 secretion induced the expulsion of *Nippostrongylus brasiliensis*, a helminth, in a mouse model that exhibits delayed worm expulsion (93). IL-25 was also found to have protective capabilities in an *ex vivo* enteroinvasion challenge model where exogenous IL-25 restored intestinal barrier functions and prevented bacterial infections (94). While the beneficiary role of IL-25 extends to homeostasis and wound healing, these features have so far been limited to the keratinocytes in the skin and the gastrointestinal epithelium.

Since its identification almost two decades ago, various studies have sought to delineate the role of IL-25 as an initiator of T2 responses. Since the seminal study by Fort and colleagues (83), in which the administration of IL-25 to mice was shown to elicit the production of T2 cytokines and induce gross eosinophilia, IL-25 has proven to be a main player in shaping allergy and asthma responses. Of clinical relevance is the association of IL-25 production with asthma exacerbations triggered by respiratory viruses (95). The induction of IL-25 by rhinoviruses in lung epithelial cells and concomitant activation of T2 inflammation was distinctly higher in samples from asthma patients (95). This finding has uncovered a role for IL-25 signalling in dampening antiviral responses, which can be targeted as a strategy for asthma treatment. IL-25 is also involved in chronic inflammation from inflammatory bowel disease (IBD) and rheumatoid arthritis (RA). In IBD, IL-25 is characterized has a pleiotropic protein, having both beneficial and negative effects depending on stage of the disease and the type of studies. Human patients with IBD have very low IL-25 levels in their serum and inflamed mucosa suggesting IL-25 presence could prove beneficial. However, IL-25-deficient mice were found to be more resilient to dextran sulfite sodium (DSS)-induced colitis, resisting up to 3 treatments before levels of inflammatory markers rose. In that study, IL-25 upregulated T2 responses from colon epithelial cells with increased IL-33, IL-6 and TNF-α levels absent in the IL-25^-/-^ (96). In RA, IL-25 has been reported to be produced in excess in synovial fibroblasts suggesting a pro-inflammatory role. Later studies found IL-25 could suppress the pathogenesis response of Th17 cells (97).

In asthma, the role of IL-25 appears to be primarily recognised as a detriment to positive health outcomes. Lipopolysaccharide (LPS), ovalbumin (OVA), and injury to the epithelium are known to activate epithelial IL-25 release (92). OVA and LPS activate IL-25 production through the MAPK p38 and JNK pathway *in vitro* (86). Airborne allergens such as cockroach extract (CE) and HDM and their associated proteases can trigger the secretion of IL-25 along with IL-33 and TSLP (6, 98). The release of IL-25 from epithelial cells in asthma also leads to overproduction of IL-4, IL-5, IL-9, IL-13, and CCL11, which then initiate recruitment of eosinophils, DCs, ILC2s, basophils, T cells, and other immune cells (86–88, 99). Among these recruited cells, IL-25 induces eosinophils through NF-κB, p38 MAPK and JNK pathways to produce chemoattractant protein-1 (MCP-1) which accelerates monocyte/macrophages recruitment, macrophage inflammatory protein-1α (MIP-1α) which can cause neutrophilic infiltration and the pro-inflammatory cytokine IL-6 and IL-8 (100, 101). Recent findings highlighted the presence of IL-17RB^+^ myeloid dendritic cells (mDCs) and IL-17RB^+^ plasmyeloid dendritic cells (pDCs) in humans (102). These newly identified DCs have potential pro-inflammatory and anti-inflammatory roles following IL-25 induction for mDCs and pDCs respectively (102).

Airway remodeling is also associated with IL-25, which is characterised by goblet cell hyperplasia, mucus hyperproduction, and enhanced angiogenesis through increased endothelial cell expression of vascular endothelial growth factor (VEGF) that activates the PI3K/Akt and Erk/MAPK pathways (28, 83). In airway remodeling, IL-25 induces the differentiation of fibroblasts into myofibroblasts and increases extracellular matrix deposition of collagen I/III and fibronectin in the lungs (80, 91). IL-25 is a major driver for airway remodeling in asthma by causing increased epithelial damage, which then maintains eosinophil and immune cell infiltration by neutrophils and T cells and creates a positive feedback loop system that can perpetuate inflammation (28, 81, 83–85). The direct link of IL-25 to fibroblast activation in inflammation is unique among the alarmin cytokines and has led to an initiative to generate potential therapeutic targets that target IL-25. Allergen-primed memory-like ILC2 cells have been demonstrated to be highly responsive to IL-25 (103). Recently, tuft cells in the airway epithelium were discovered to be a key source of IL-25 and were responsible for driving the allergic inflammatory response together with cysteinyl leukotrienes (104, 105).

A similar cell type was found in murine synovial epithelium named solitary chemosensory cells (SCCs). This suggests a similar activation of these cells by their taste receptor could regulate the immune response to allergens. It is currently believed a similar type of IL-25 positive chemosensory cell exist in the human nasal cavity which would increase the relevance of IL-25 in asthma even further (91).

As found for TSLP, there is limited information about how IL-25 is secreted from cells despite extensive knowledge of signalling pathways that trigger its production. Taken together, the pro-inflammatory effects of IL-25 in allergic airway inflammation is in marked contrast to its role in protection against helminth infection.

### Role of interleukin-33 (IL-33) in allergic airway inflammation

IL-33 was first identified in 2003 as “nuclear factor from high endothelial venules” (NF-HEV) before being assigned to the IL-1 family in 2005 (27). IL-33 is constitutively expressed and chromatin-associated in endothelial cells in large blood vessels throughout the body, as well as in epithelial cells, with a higher abundance in mucosal epithelial cells in contact with the external environment, including gastrointestinal and bronchial epithelia (32, 106, 107). IL-33 is highly expressed in the nuclei of various cells in the steady state and contributes to tissue homeostasis and responses to environmental stimuli (106). Its constitutive expression in various tissues is attributed to its prevalence in endothelial cells from blood vessels along the vascular tree (106). Nevertheless, IL-33 expression is further increased in response to inflammatory cues such as necrotic cell death, mechanical and oxidative stress (108). In addition, IL-33 can be proteolytically activated by allergen and calpain proteases, which leads to increased processing and release from damaged airway epithelial cells (108). Interestingly, the half-life of IL-33 is brief due to oxidation (within minutes of its release), suggesting that it may serve as a “molecular clock” that limits its range and duration of activity in response to airway stimuli (109).

As a pleiotropic protein, IL-33 is both a pro-inflammatory cytokine and an intracellular transcriptional factor with regulatory properties. Lacking a signal peptide, IL-33 is bound directly to nuclear histones and is released directly from cell nuclei upon damage to the epithelium, so it is not released under apoptotic conditions but instead through necrosis (63, 110). Its intranuclear localisation may be important for prevention of inappropriate secretion, as IL-33 overexpressing transgenic mice are highly susceptible to systemic inflammation (111). The genetic sequences encoding *IL33* and IL-1 receptor-like 1 *(ILRL1*), which encodes ST2, are amongst the few genes associated with asthma (112). A rare variant in *IL33* correlates with low eosinophil counts and reduced risk of asthma in Europeans. Interestingly, the mutation induces truncation of the C-terminal 66 amino acids, which does not affect the intracellular localisation of IL-33 (112).

Unlike the activating effects of caspase-1 on IL-1β and IL-18 during apoptosis, caspase-1 deactivates IL-33 upon its cleavage. Caspase-3 and caspase-7 were also found to inactivate IL-33 which suggests caspases possibly act as an anti-inflammatory protective layer to limit the effects of IL-33 *in vitro* (113). Currently, studies suggest that the bioactivity of IL-33 is mediated through interaction with activated neutrophil serine proteases, elastase and cathepsin G, that cleave intact IL-33 into shorter highly active forms (99, 114–116).

Upon release, IL-33 binds to a heterodimeric cell surface receptor composed of the selectively expressed receptor IL1RL1/ST2 in combination with the IL-1R accessory protein (IL-1RacP), which is ubiquitously expressed (117). ST2 was first discovered on Th2 cells and mast cells (118–120), and since then studies have revealed its constitutive expression on most immune cells which include ILC2 cells, eosinophils, basophils, natural killer cells, NKT cells, Treg cells, cytotoxic T cells and activated Th1 cells (32, 63, 78, 99, 106, 107, 113, 115, 116, 121–129). In ILC2 cells, IL-33 induces expression of OX40L serves a critical role in Treg and Th2 cell expansion which leads to type 2 pulmonary inflammation (130).

**Figure 6:**
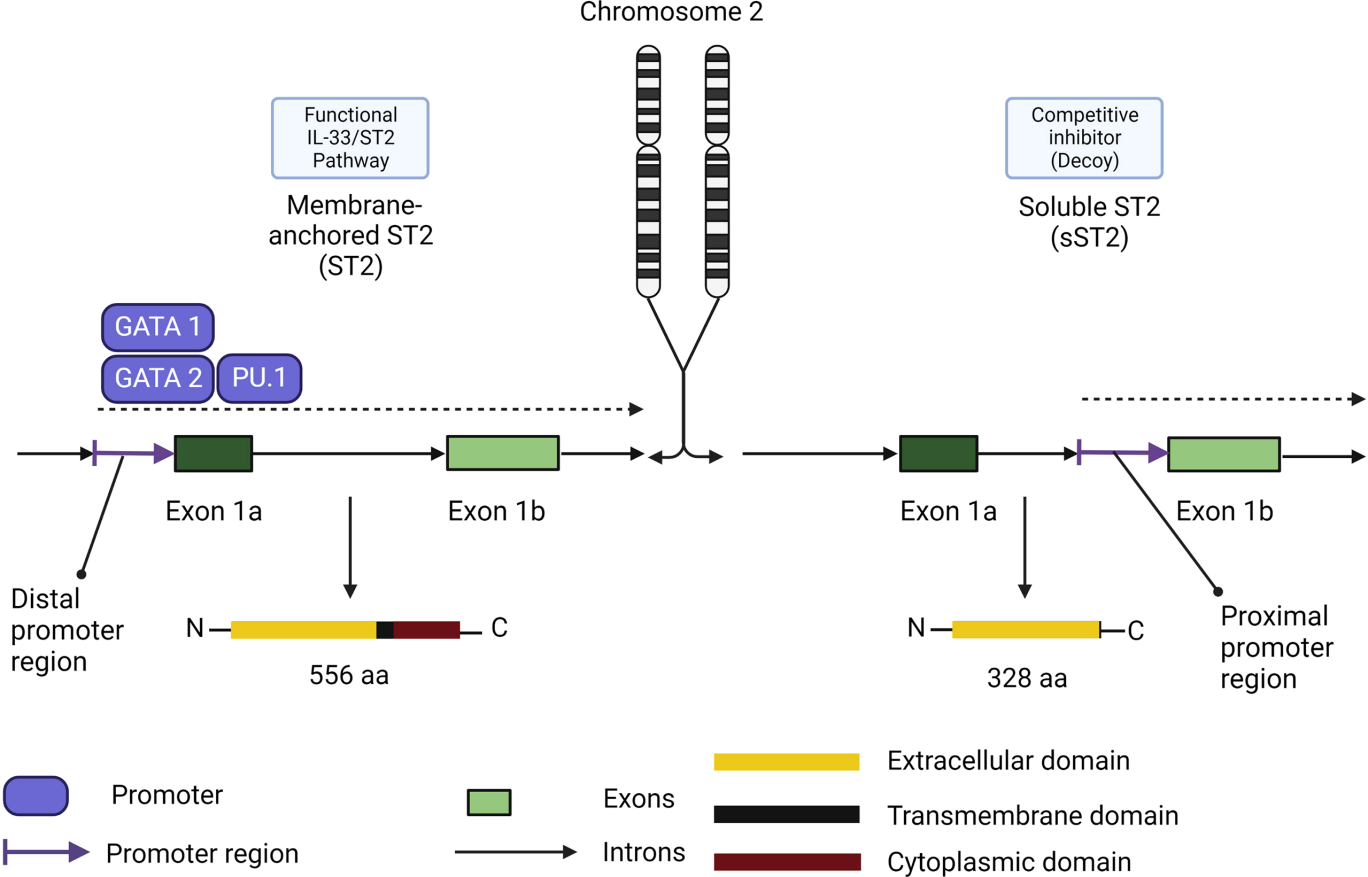
Membrane-anchored ST2/soluble ST2 isoforms functional differences in role and expression. Membrane-anchored ST2 (ST2) expression is controlled by the distal promoter region through the promoters GATA1, GATA2 and PU.1. ST2 comprises the functional receptor in the IL-33/ST2 signaling pathway. Soluble ST2 (sST2) expression is controlled by the proximal promoter region. Its promoters remain to be elucidated and the isoform share the same extracellular domain which makes sST2 a competitive inhibitor of ST2 in the IL-33/ST2 signaling pathway.

Similar to TSLP and IL-25, the receptor for IL-33 possesses two main isoforms (**Figure 6**). The membrane bound ST2 forms a heterodimer with IL-1RAP and activates the ST2/IL-33 signaling pathway, while the soluble form of ST2 (s)ST2 acts as a decoy to bind free IL-33 and prevent IL-33/ST2 signaling pathway activation (131). Another human isoform of ST2 exists (ST2V), although little information is currently available about its functionality. ST2V is highly expressed in the stomach, small intestine and colon, and is regulated by alternate promoter binding sites (132).

sST2 acts as a negative regulator of IL-33/ST2 signaling and dampens the pro-inflammatory effects of IL-33. The function of sST2 as a down regulator of IL-33 signaling was first discovered in a murine asthma model (131), BALB/C mice were challenged with OVA to confirm the increasing expression of both ST2 and sST2. They later proceeded to pre-treat murine thymoma EL-4 cells stably expressing membrane-bound ST2 with sST2 which supressed the production of IL-4, IL-5 and IL-13 (131), but the exact location of sST2 production was not found at the time. We now know that sST2 in humans is produced by lung, heart, kidney, small intestine cells, and macrophages (133). sST2 production is also increased by IL-1β and TNF-α in human lung epithelial cells as well as cardiac myocytes *in vitro*, and IL-33 itself can induce production of sST2 by mast cells (134). For its counterpart, ST2 expression can be enhanced by IL-33 on eosinophils, Th9 cells, innate lymphoid cells, and regulatory T cells (106, 122, 135). Macrophage ST2 expression is enhanced by IL-4 and IL-13 while basophil ST2 expression is regulated by IL-3 stimulation (136–140). Finally, Th2 cell ST2 expression is dependent on GATA3 signaling and can be enhanced by IL-6, IL-1, TNF-α or IL-5 (119, 141). Other than sST2, ST2 has two other forms of down-regulation. Downregulation may occur through the ubiquitin-proteasome system which degrades ST2 internally, and TIR8, also known as SIGIRR which was found to disrupt the ST2/IL-1RacP dimer (142–145). When IL-33 binds to ST2 and not the ST2 decoy, it triggers ST2 to form a heterodimer with IL-1RAP which recruits MyD88 as shown in **Figure 6**. MyD88 binding is followed by IL-1R-associated kinase activation (IRAK) which activates TNF receptor-associated factor 6 (TRAF6). TRAF6 then activates mitogen-activated protein kinase (MAPK) and the inhibitor of nuclear factor-κB (NF-κB) kinase (IKK) complex. MAPK subsequently activates activator protein 1 (AP-1) while IKK liberates NF-κB which both leads to T2 cytokine upregulation (106, 146). While the pathways of IL-33/ST2 appear complete, there are still some unknowns when it comes to the downstream signaling. Currently, AP-1 is hypothesized to have a pathway independent of MAPK or NF-κB and a TRAF-6 independent pathway through MAPK which has not been elucidated yet seems to also be able to induce T2 cytokine (145). Studies on Tregs from the colon have also found a pathway involving GATA3 and *Foxp3* that culminates in promotion of ST2 receptor on the surface of Tregs and Th2 cells irrespective of the presence of inflammation (147).

In short, IL-33/ST2 signaling is regulated by ST2 expression of its two main isoforms, the membrane-bound ST2 (ST2) and its free soluble ST2 (sST2). It is quite likely that, it is the actual ratio of ST2/sST2 in a given area that determines IL-33/ST2 signaling outcomes. This ratio can itself be influenced by the ubiquitin-proteasome system, the type and number of cells in the environment, certain cytokine levels such as, IL-4, IL-5, IL-6 or IL-13. The inhibitory effects of sST2 has led companies to look further into creating competitive inhibitor to the heterodimer ST2/IL-1RAcP to sequester IL-33 with the aim to reduce Th2 inflammation induced by IL-33.

IL-33 is capable of directly influencing both adaptive and innate immune cells to promote inflammation. In the innate immune system, IL-33 promotes the initiation of inflammation by interacting with ILC2s, basophils, and NK cells to induce secretion of T2 cytokines like IL-4, IL-5, and IL-13. In turn, these pro-inflammatory cytokines activate the adaptive immune system, recruit eosinophils, and initiate the process of airway inflammation and remodeling. In the adaptive immune system, IL-33 induces chemotaxis of Th2 cells and interacts with them to promote their proliferation and enhance their survival (125, 148). The effects of IL-33 has been posited to possibly surpass IL-25 in causing airway hyperreactivity by expanding the ILC2 population and inducing IL-13 to a greater degree than its counterpart based on studies in mice deficient in either IL-25 or IL-33 (99). Mice deficient in IL-33 have also proven to be resilient to HDM-induced allergic rhinitis as well as ragweed pollen challenges (99, 149, 150). T2-independent airway hyperreactivity is also linked to IL-33, where it was shown that activated NKT cells could induce alveolar macrophages to produce IL-33, which then induced IL-13 production by ILC2s and activated NKT cells, creating a positive feedback loop (29, 117, 125). This innate T2 cytokine response was also observed in a mouse model examining danger signals (63). In this study, ATP release from damaged cells induced IL-33 production in mouse lungs, which led to the activation of ILC2s, which then produced IL-5 and IL-13 to initiate a T2 response (63). Studies on chronic inflammation from smoking and COPD suggests the continuous inflammatory signals increases tissue availability of IL-33 which then contributes to the state of inflammation in a feedback loop (107). Other studies on IL-33-induced airway hyperreactivity demonstrated a link between IL-33 and mucus overproduction, goblet cell hyperplasia, and eosinophilia, as well as elevated chemokines and cytokines in the lungs (106). From these findings, it has been determined that IL-33 is a central mediator in inflammation in both the innate and adaptive immune systems.

In addition, IL-33 can synergise with both IL-25 and TSLP to promote cell migration and increase ILC2 activation (116). In homeostasis, the role of IL-33 has been partially characterised but less well understood. Studies suggest strategically positioned Tregs that express a high level of IL1RL1 receptors are able to bind IL-33 and activate highly anti-inflammatory Tregs, which then secrete IL-10 (117). Additionally, IL-33 can promote a subset of ILC2s and DCs that enhance Treg proliferation. These findings suggest a model where excessive IL-33 can be intercepted by these anti-inflammatory pathways to prevent propagation in local immunity.

## Perpetrators of the asthma inflammatory response: Synergism or redundancy?

As mentioned above, TSLP, IL-25 and IL-33, are designated as alarmin cytokines because their release from microbial, pollutant or allergen-perturbed epithelia heralds the onset of inflammatory responses in asthma. Studies on the biology of these cytokines indicate a role in tissue homeostasis under steady-state conditions, which switches to the induction of inflammatory mediators upon dysregulation or triggering by environmental cues.

The ability of alarmin cytokines secreted by epithelial cells to mediate similar downstream inflammatory responses indicates a degree of redundancy in the production of T2 cytokines, biomarkers and pathophysiological manifestations characteristic of asthma. Consequently, studies have been carried out to identify how individual alarmin cytokines contribute to the asthma inflammatory responses. Observations from alarmin cytokine/cytokine receptor-deficient mouse experiments indicate that individual alarmins can elicit the infiltration of different of immune cells and their corresponding responses depending on the nature of the allergen. For instance, the accumulation of eosinophils and ILC2 cells were significantly reduced in the BALF of TSLPR-deficient mice in an innate model of papain-induced airway inflammation (151). This corresponded with reduced levels of IL-5, IL-13 and reduced goblet cell hyperplasia (151). Furthermore, the magnitude of T2 inflammation, indicated by the numbers of eosinophils and ILC2s in the BALF of papain-challenged basophil-specific TSLPR-deficient mice, was significantly higher compared to lymphoid cell-specific TSLPR-deficient mice (151). In contrast, IL-5 and IL-13 were mainly produced by CD4^+^ T cells and not ILC2 cells in an OVA-induced atopic march model. Also, reduced T2 responses exhibited by conventional DC-specific TSLPR-deficient mice and lymphoid cell-specific TSLPR-deficient mice in this model indicate that TSLP acts directly on DCs and CD4^+^ T cells (151). However, a recent study showed that individual ablation of TSLP, IL-25, or IL-33/ST2 has no effect on the development of T2 dependent inflammation driven by IL-4 and IL-13; while blockade of all three cytokines greatly reduced eosinophilia in mouse models of helminth-induced T2 inflammation and chronic allergic inflammation (152). These results reflect specific mechanisms which may be utilised by alarmin cytokines exhibiting a degree of redundancy and facilitating T2 inflammation during early and late stages of allergic asthma.

The deletion of IL-33 has also been shown to impact the infiltration of eosinophils, neutrophils and CD4^+^ T cells in chronic HDM-induced lung inflammation, which indicates its critical role during the onset of asthmatic inflammation (153). In a mouse model of asthma induced by prolonged HDM, targeting IL-33 using a neutralizing antibody normalised established eosinophilic, neutrophilic, and ST2^+^CD4^+^ T-cell infiltration and improved remodeling of both the lung epithelium and parenchyma (146). Treatment with anti-IL-33 also restored the presence of ciliated cells over mucus-producing cells and decreased myofibroblast numbers, even in the context of continuous allergen exposure, indicating a key role for IL-33 in lung remodelling (146).

IL-33, like IL-25, has been found to be associated with virus-associated asthma exacerbations. However, unlike IL-25 that promotes virus-linked asthma exacerbations by amplifying T2 responses, IL-33 can facilitate this process by hampering innate and adaptive Th1 and cytotoxic responses (154). Targeting IL-33 secreted by bronchial ciliated cells and type II alveolar cells resulted in the restoration of Th1 cytokine and chemokine levels as well as IFN-β expression in structural and immune cells (154). The mechanisms through which IL-33 and IL-25 differentially counter anti-viral responses suggest that alarmin cytokines can promote asthma severity culminating from various viral infections, which can be harnessed as treatment strategies.

Studies indicate that IL-25, like IL-33 and TSLP, has a prime position in promoting T2 asthma. Results from a recent study show that targeting IL-25 can induce a more selective phenotype compared to the broader effects of IL-33 (153). In a chronic HDM-induced asthma model, IL-17RB-deficient mice exhibited altered tissue remodelling and airway hyperresponsiveness, but not eosinophilia and mucus production (153). Also, IL-25 can function in an autocrine manner by promoting epithelial cell expression of TSLP (155). Data from mouse pre-clinical studies indicate that targeting IL-25 using a neutralising antibody resulted in reduced AHR, which was apparent after antibody administration during ongoing T2 inflammatory responses in the lungs (156).

Multiple studies described here and elsewhere specify that the activities of TSLP, IL-25 and IL-33 serve as a bridge between the epithelium and downstream airway inflammatory responses.

Observations from several studies have established that the predominance of alarmin cytokines in promoting allergic airway inflammation may depend on differing sensitization and challenge protocols adopted in mouse preclinical studies. Therefore, a logical approach would be to assess whether alarmin cytokines act synergistically and if this can be targeted as a strategy to alleviate airway inflammation and asthma exacerbation. As expected, however, varying results have been obtained from co-targeting studies using mouse pre-clinical models. For instance, in a virus-induced HDM asthma exacerbation study, targeting TSLP did not impact anti-ST2-mediated reduction of airway inflammation (154). Instead, TSLP synergised with IL-33 to mediate AHR at late time-points, indicating the relevance of the IL-33/ST2 axis rather than TSLP in facilitating airway inflammation at this late stage of inflammation.

Results from a chronic HDM–induced allergic lung inflammation study have indicated that combined targeting of TSLP, IL-25 and IL-33 using antibodies does not further decrease established inflammation or fibrosis (152). However, treatment of TSLP/IL-33 double-knockout mice with anti-IL-25 antibody during the initiation of allergic airway inflammation reduced inflammation, mucus production, and lung remodeling in the chronic phase (152). Amongst the many conclusions which can be drawn from this study it is evident that there is a degree of redundancy in the roles of TSLP, IL-25 and IL-33 in maintaining downstream T2 inflammatory responses and pathology. In summary, co-targeting alarmins during the early inflammation phase before the establishment of chronicity may alleviate subsequent allergic airway manifestations.

## Nipping the asthmatic cascade in the bud: Targeting alarmin cytokines as an asthma therapeutic strategy

With the increasing interest in alarmin cytokines and their roles in inflammatory cascades in asthma, a growing number of studies developing strategies to neutralize their upstream effects on asthma have been ongoing for the last two decades. A multitude of humanized monoclonal antibodies for asthma treatment are currently undergoing clinical development, which include tezepelumab (targeting TSLP) as well as etokimab, itepekimab, astegolimab (AMG 282), torzorakimab, and melrilimab (GSK_3772847_) for IL-33 (**Table 1**). The effects of tezepelumab are exerted by binding to circulating or interstitial TSLP and preventing its interaction with the TSLP receptor complex (157–162, 166, 167). Tezepelumab has already successfully completed phase II and III clinical trials, with results indicating a reduction in rates of asthma exacerbations, improved FEV_1_, and a reduction of T2 inflammatory biomarkers such as IL-5, IL-13 periostin, and IgE in both T2 high and T2 low individuals with severe asthma, and without an increase in adverse effects over those of placebo (157–162, 166). Results from the phase 2 study PATHWAY trial with tezepelumab went under *post hoc* analysis which indicated that annual asthma exacerbation rates were reduced irrespective of blood eosinophils counts, baseline body mass index (BMI) or participants IgE levels (Th2-low asthma patients) (168), suggesting that tezepelumab has broad efficacy across different phenotypes of asthma (157, 159, 160). Results from the NAVIGATOR trial using tezepelumab suggest that targeting TSLP in patients with severe, uncontrolled asthma may prevent exacerbations as well as improve lung function based on improved prebronchodilator FEV_1_ values (169). *Post hoc* analysis from these studies also revealed tezepelumab reduced blood levels of IL-5, IL-13, periostin, and thymus and activation-regulated chemokine (TARC) (157, 159, 169). Observations from these clinical trials suggest that tezepelumab could be a prospective therapeutic option to prevent severe asthma exacerbations by targeting cytokine signals further upstream at the level of the bronchial epithelium.

**Table 1:** Clinical trials of drugs targeting alarmins in asthma.

Treatment	Monoclonal antibody	Current clinical phase	Intended application
**Anti-TSLP**	Tezepelumab (AMG157)	Phase III in asthma	Asthma (36, 157–163)
**Anti-IL-33**	Etokimab (ANB 020)	Phase II in eosinophilic asthma	Asthma, allergy, atopic dermatitis, chronic rhinosinusitis with nasal polyps (study postponed following lack of outcomes at week 8)
	Itepekimab (SAR440340)	Phase II in asthma	Asthma (164) (study ongoing)
	Tozorakimab (MEDI3506)	Phase II in asthma	Asthma (study completed, data under analysis)
**Anti-IL-33 receptor (IL1RL1/ST2)**	Astegolimab (AMG282)	Phase II in asthma	Asthma, chronic rhinosinusitis with nasal polyps (165)
	Melrilimab (GSK3772847)	Phase II in asthma	Asthma (study ongoing)

However, the SOURCE study, a phase 3 multicenter clinical trial across seven countries with 150 participants, showed different results with tezepelumab (162). The SOURCE study found that tezepelumab had no effect in improving dose reduction of oral corticosteroids compared to placebo. In addition, participants with a blood eosinophil count > 150 cells per μL had an observable improvement with tezepelumab (162). Thus, the findings from the SOURCE study contradict earlier results from the PATHWAY study indicating that asthma exacerbations were reduced irrespective of blood eosinophil counts. Further studies on tezepelumab are required to determine the nature of this discrepancy and identify asthmatic populations best suited for its use as a treatment.

While prospects are promising for prevention of TSLP-mediated inflammation through tezepelumab, therapies against the other two alarmins, IL-25 and IL-33 are currently lagging as effective treatments in asthma. Despite evidence that blockade of IL-25 prevents airway hyperresponsiveness in numerous mouse models of allergic asthma, such outcomes have not been reproduced in clinical trials (116, 127, 152). Commercially available biologics for IL-25 exist in the form of brodamulab targeting the IL-25 receptor, IL-17 receptor A (IL-17RA), although this is limited to treating plaque psoriasis (168). Another monoclonal antibody against IL-25 with encouraging early clinical data is bimekizumab, which selectively neutralizes IL-17A and IL-25 (IL17-F) (170). Still, like brodamulab, the extent of bimekizumab use is currently limited to the treatment of psoriasis, psoriatic arthritis, and ankylosing spondylitis, according to early clinical data. Bimekizumab has been proposed as a biologic treatment for asthma, although this remains uncertain since this biologic was developed only recently.

IL-33 biologics have shown limited progress in exploring their use for asthma treatment, despite having been tested in more clinical trials than those of IL-25. Current therapeutic strategies aim to neutralize IL-33 and prevent extensive interaction with its associated receptor ST2 by three strategies: neutralizing IL-33 directly, deploying soluble decoy receptors of ST2 to competitively inhibit the receptor, or targeting the ST2 receptor (29, 136, 171). The mechanism of action of astegolimab falls within the first strategy, consisting of a human mAb that binds to IL-33 and prevents the interaction of IL-33 to ST2, which is currently under phase I trial investigation (166, 172).

Results from the recent ZENYATTA study indicate that astegolimab, a human IgG_2_ mAb which targets IL-33 receptor, ST2, reduces asthma exacerbation rates in both eosinophil-high and -low patients with inadequately controlled, severe asthma (165). This study demonstrated that astegolimab reduced the annualized asthma exacerbation rate by 43% relative to placebo in patients with severe asthma for both eosinophil-low and eosinophil-high patients, suggesting that this treatment may benefit a wider spectrum of asthmatics than only those with elevated blood eosinophils (165). Another biologic that directly targets IL-33, itepekimab, was recently found to improve lung function and lower the incidence of uncontrolled asthma events in a monotherapy cohort of a phase II trial in asthma (164). Itepekimab was found to improve FEV_1_, asthma control, and quality of life compared with placebo in patients with moderate to severe asthma compared with placebo (164). With both astegolimab and itepekimab continuing in clinical trials, there is still opportunity for the development of effective IL-33 biologics for treating asthma.

Other studies are under way now to investigate the efficacy of targeting the IL-33 pathway in asthma including melrilimab, which inhibits ST2, and tozorakimab, a phase II study with anti-IL-33 which has been completed and the data are currently under analysis.

Overall, the upstream positions of alarmin cytokines in asthma render them as prime candidates for antibody treatment, and these findings suggest that their inhibition may provide a useful approach to manage outcomes in asthma. Current studies are still under way to optimize treatment strategies for patients with asthma, while tezepelumab has received FDA approval for its use in severe asthma treatment and has already been launched for clinical use in the USA. In the meantime, other downstream effector cytokines, such as IL-4, IL-5, and IL-13, remain the current best available targets for biologic treatment of severe asthma with their associated biologics such as dupilumab, an IL-4 receptor antagonist, benralizumab, an IL-5R-binding antibody, reslizumab and mepolizumab, an IgG_1_ and an IgG_4_ neutralizing IL-5 antibody, and omalizumab, an anti-IgE antibody which binds to the Fc region of free IgE (173–175).

## Summary

Research on epithelial cell alarmin cytokines as frontline mediators of the inflammatory response in asthma is still ongoing, as is the development of new biologic therapies that target this group of cytokines. The promising outcomes using tezepelumab as a new therapy for both T1 and T2 inflammation in asthma suggest that this could be a very useful treatment for severe asthma. Future directions lie in understanding more about the expression profiles of sfTSLP and lfTSLP in epithelial cells throughout the body, and how tezepelumab affects the activities of these two isoforms during inflammation.

## Author contributions

MD, IO, and PL wrote the manuscript. MD and IO performed the literature review and data collection. MD prepared **Table 1** and Figures. PL revised the manuscript. All authors contributed to the article and approved the submitted version.

## Funding

MD, IO and PL were funded by AstraZeneca Canada (ESR-20-20575). The funder had no role in the study design, data collection and analysis, decision to publish, or preparation of the manuscript.

## Acknowledgments

The authors would like to thank Kevin Johns, Jenny Jagers, and Alain Gendron from AstraZeneca Canada for helpful discussions regarding topics covered in this review. All figures displayed in this article were created with BioRender.com.

## Conflict of interest

PL reports grants from Natural Science and Engineering Research Council of Canada, and Synergy Respiratory and Cardiac Care, and personal fees from GlaxoSmithKline Canada, AstraZeneca Canada, and Synergy Respiratory and Cardiac Care, Canada, outside this submitted work.

The remaining authors declare that the research was conducted in the absence of any commercial or financial relationships that could be construed as a potential conflict of interest.

## Publisher’s note

All claims expressed in this article are solely those of the authors and do not necessarily represent those of their affiliated organizations, or those of the publisher, the editors and the reviewers. Any product that may be evaluated in this article, or claim that may be made by its manufacturer, is not guaranteed or endorsed by the publisher.
